# Unstability of 4-CMC in human serum specimen

**DOI:** 10.1007/s11419-018-0455-4

**Published:** 2018-11-19

**Authors:** Karolina Nowak, Paweł Szpot, Marcin Zawadzki

**Affiliations:** 1 Department of Forensic Medicine, Wroclaw Medical University, 4 Jana Mikulicza-Radeckiego Street, 50-345 Wrocław, Poland; 2 Institute of Toxicology Research, 45 Kasztanowa Street, 55-093 Borowa, Poland

Dear Editor,

Determination of new psychoactive substances (NPS) and interpretation of the results of research on them is a challenge for both clinical and forensic toxicologists. Among others, scientists undertake to develop methods for rapid determination of NPS in biological material or examine their pharmacokinetic and pharmacodynamic properties. Adequately quick determination of substances in biological material may help in choosing the right treatment method and thus protect patients from serious health consequences or even death. On the other hand, post-mortem toxicological analysis may help to determine the cause of death and prevent similar cases in the future. The results of post-mortem examinations are influenced by thanatochemical processes. Post-mortem changes negatively affect the stability of the substances in biological material, especially when the body is in a state of progressive putrefaction. Researchers [1–5] undertook studies into the stability of NPS in various biological matrices, including synthetic cathinones such as 4-methylmethcathinone (4-MMC), 4-ethylmethcathinone (4-EMC), and 3,4-dimethylmethcathinone (3,4-DMMC). However, none of the abovementioned studies examined the stability of 4-chloromethcathinone (4-CMC) in biological material. The authors of this study noticed the need to carry out research in this area because 4-CMC is highly unstable at 4 °C. The significance of research into this substance is further reinforced by the European Drug Report 2018 [6], according to which in 2016 in Europe 4-CMC was the second most frequently confiscated cathinone and the one seized in the largest amount (890 kg).

This letter presents a study of 4-CMC stability in a blood serum sample without any preservatives (obtained from a person taking 4-CMC), stored at 4 °C.

The 4-CMC, (1-(4-chlorophenyl)-2-(methylamino)-1-propanone), also known as clephedrone, was first introduced to online sales in 2014 [7]. Although this substance has been available on the black market for several years, the results of research on, among others, its pharmacokinetic properties, toxic doses, effects of use, or addictive potential remain insignificant. Information on patterns of use are usually passed between users via online forums [8]. Tomczak et al. [8] determined that the blood concentration of 4-CMC in non-fatal cases ranged from 1.3 to 75.3 ng/mL (*n* = 9) and in fatal cases from 56.2 to 1870 ng/mL (*n* = 5).

**Fig. 1:**
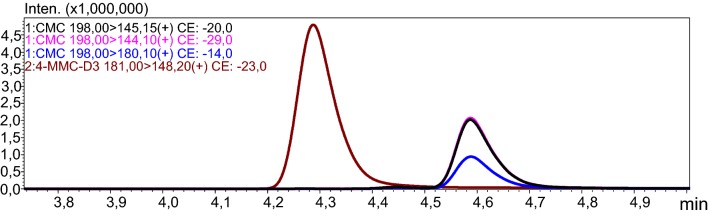
MRM for 4-CMC and IS from first analysis of serum sample

**Fig. 2:**
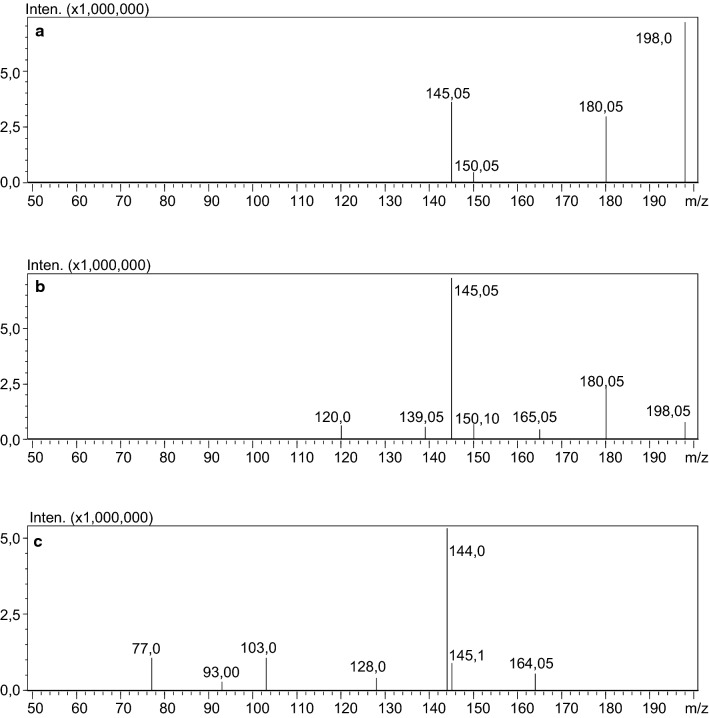
Mass spectra of 4-CMC; collision energy: **a** 10; **b** − 20; **c** − 35

The discussed case concerns a 27-year-old incarcerated man. The reason for ordering toxicological tests is unknown. There is also no information about the medical or drug history of the man. The material submitted for testing in the form of blood serum was screened for numerous drugs. The serum was extracted with ethyl acetate from alkaline medium (pH 9). The analysis was carried out using the technique of ultra-high-performance liquid chromatography-tandem mass spectrometry (UHPLC-QqQ-MS/MS). Calibration range 0.5–100 ng/mL; LOQ: 0.5 ng/mL. 4-MMC-*d*_3_ was used as an internal standard. Figure 1 shows the chromatogram obtained from first analysis with MRM transitions for 4-CMC and IS. Figure 2 shows mass spectra of 4-CMC.

At the time of the first analysis (day 0), the 4-CMC concentration was 11.5 ng/mL. Subsequent analyses were conducted on the days 3, 8, 16, 27, 47, and 113. The sample was stored at 4 °C throughout the testing period. Table 1 shows 4-CMC concentrations on subsequent days.

**Table 1:** Concentrations of 4-CMC in serum sample on subsequent days

Days	Concentration (ng/mL)	Comparison with the concentration of first analysis (%)
0	11.5	100
3	4.0	34.8
8	2.6	22.6
16	2.1	18.3
27	1.5	13.0
47	0.5	4.3
113	0.2 (< LOQ)	1.7

The tests demonstrated that 4-CMC was unstable in a blood serum sample stored at 4 °C. As early as 3 days after the first quantitative measurement, the analyte concentration dropped by 65% (11.5 ng/mL at day 0 vs. 4.0 ng/mL at day 3). The analyte was considered stable if the concentration differences were within ± 20% of the initial concentration. On day 47, the analyte concentration slightly exceeded LOQ and on the last day of the study, i.e., on day 113, the concentration was well below LOQ.

To the best of our knowledge, so far no studies have been published on the stability of 4-CMC in biological material. A review of the available literature discussing the stability of cathinones, which include 4-CMC, confirms that some substances from this group are unstable in biological matrices.

Glicksberg and Kerrigan [3] carried out research on the stability of 22 cathinones in whole blood. Among the secondary amines, the lowest stability at 4 °C was shown by 4-fluoromethcathinone (4-FMC, flephedrone) [*T*_1/2_ (half-life) = 13 days] compared with the other cathinones from this group, ranging from *T*_1/2_ = 2.7 months for 4-EMC to *T*_1/2_ = 5.9 months for methedrone (*n* = 11). For methylone, which is a secondary amine MD-substituted (methylenedioxy-substituted) cathinone, *T*_1/2_ amounted to 9.6 months at 4 °C and for cathinones belonging to the group of tertiary amines *T*_1/2_ amounted to, respectively, 10 months (naphyrone) and 15 months (MPBP-4-methyl-α-pyrrolidinobutiophenone). The 4-FMC showed instability (20% decrease) at 4 °C in 4 days. While studying stability of, among others, mephedrone, Johnson and Botch-Jones [4] showed that after 14 days at 4 °C the concentration of the substances decreased by, respectively, more than 50% in whole blood and by 31% in serum. On the other hand, Da Cunha et al. [2] showed a decrease in the mephedrone concentration in whole blood at 4 °C by about 40% after 90 days. For comparison, benzedrone detection was possible for up to 60 days at 4 °C (for 30 ng/mL). Busardò et al. [9] examined the stability of mephedrone in ante-mortem (*n* = 10) and post-mortem (*n* = 10) blood samples. In ante-mortem samples stored with NaF/KOx (sodium fluoride/potassium oxalate) at 4 °C, the concentration of mephedrone decreased after 1 week and after 31 days by, respectively, 28.3% and 63.6% while in samples with EDTA (ethylenediaminetetraacetic acid) it decreased by, respectively, 30.5% and 68.5%. In ante-mortem samples without preservatives, the concentration of mephedrone decreased after 1 week by 50% and after one month by 71.3%. The concentration of mephedrone was similar in post-mortem samples. After 1 week and after 31 days, the analyte concentration decreased by, respectively, 31% and 64.2% (with NaF/KOx), 38.1% and 68.7% (with EDTA), and 48.9% and 75.8% (without preservatives). It should be emphasized that most of these studies used a pretreated blood sample with added synthetic cathinones, while our study used a real serum sample.

Researchers [10, 11] also pay attention to the significant effect of pH on the stability of synthetic cathinones. Glicksberg and Kerrigan [10] showed a high dependence between cathinone stability and urine pH. Synthetic cathinones were more stable in acidic urine (pH 4) in comparison to alkaline urine (pH 8). Tsuijkawa et al. [11] demonstrated the stability of seven analogs of methcathinone in acidic solution (pH 4) and increased degradation of the tested cathinones in solutions with neutral (pH 7) and alkaline pH (pH 10 and 12). Degradation rates of cathinones increased with increasing of pH, but also varied with chemical structures. In addition, post-mortem blood pH level decreased in vivo (in corpses) and in vitro (in tubes). Donaldson and Lamont [12] measured pH changes in human blood and post-mortem rat blood stored in EDTA tubes and pig and rat blood stored in corpses. After 96 h pH decreased in pig and rat blood from 7.45 (ante-mortem) to 5.1. The pH of human blood and post-mortem rat blood stored in a tube decreased from 7.4 to 7.1 after 96 h. Researchers [12] consider that the rapid decrease in blood pH level in corpses is related to the accumulation of ions and metabolites that build up in a corpse due to autolysis. In this regard, pH value of serum specimens in physiological range could have an impact on stability of cathinones, including 4-CMC.

In cases of suspected cathinone use, including 4-CMC, it is important to perform determinations as soon as possible after collection. In such cases, storage of biological material samples at 4 °C seems inappropriate. Perhaps 4-CMC has better stability in biological matrices at − 20 °C. However, the authors stress the need for further research on a larger study group to confirm these observations. Based on current research into the stability of cathinones, it would be important to perform studies on the stability of 4-CMC and other isomers taking into account different biological matrices, temperatures, pH, and preservatives. The authors also suggest to determine degradation products of 4-CMC in a stability study to check for changes in concentrations of those products.
